# Myeloid AMPK signaling restricts fibrosis but is not required for metformin improvements during CDAHFD-induced NASH in mice

**DOI:** 10.1016/j.jlr.2024.100564

**Published:** 2024-05-17

**Authors:** Julia R.C. Nunes, Conor O’Dwyer, Peyman Ghorbani, Tyler K.T. Smith, Samarth Chauhan, Victoria Robert-Gostlin, Madison D. Girouard, Benoit Viollet, Marc Foretz, Morgan D. Fullerton

**Affiliations:** 1 Department of Biochemistry, Microbiology and Immunology, Faculty of Medicine, Centre for Infection, Immunity and Inflammation, Ottawa Institute of Systems Biology, University of Ottawa, Ottawa, ON, Canada; 2 Université Paris cité, CNRS, Inserm, Institut Cochin, Paris, France; 3 Centre for Catalysis Research and Innovation, University of Ottawa, Ottawa, ON, Canada

**Keywords:** non-alcoholic steatohepatitis, NASH, AMPK, macrophage, liver, immunometabolism, fibrosis, metformin

## Abstract

Metabolic programming underpins inflammation and liver macrophage activation in the setting of chronic liver disease. Here, we sought to identify the role of an important metabolic regulator, AMP-activated protein kinase (AMPK), specifically within myeloid cells during the progression of non-alcoholic steatohepatitis (NASH) and whether treatment with metformin, a firstline therapy for diabetes and activator of AMPK could stem disease progression. Male and female *Prkaa1*^*fl/fl*^*/Prkaa2*^*fl/fl*^ (Flox) control and Flox-*LysM*-Cre^+^ (MacKO) mice were fed a low-fat control or a choline-deficient, amino acid defined 45% Kcal high-fat diet (CDAHFD) for 8 weeks, where metformin was introduced in the drinking water (50 or 250 mg/kg/day) for the last 4 weeks. Hepatic steatosis and fibrosis were dramatically increased in response to CDAHFD-feeding compared to low-fat control. While myeloid AMPK signaling had no effect on markers of hepatic steatosis or circulating markers, fibrosis as measured by total liver collagen was significantly elevated in livers from MacKO mice, independent of sex. Although treatment with 50 mg/kg/day metformin had no effect on any parameter, intervention with 250 mg/kg/day metformin completely ameliorated hepatic steatosis and fibrosis in both male and female mice. While the protective effect of metformin was associated with lower final body weight, and decreased expression of lipogenic and *Col1a1* transcripts, it was independent of myeloid AMPK signaling. These results suggest that endogenous AMPK signaling in myeloid cells, both liver-resident and infiltrating, acts to restrict fibrogenesis during CDAHFD-induced NASH progression but is not the mechanism by which metformin improves markers of NASH.

## Graphical abstract

**Figure:**
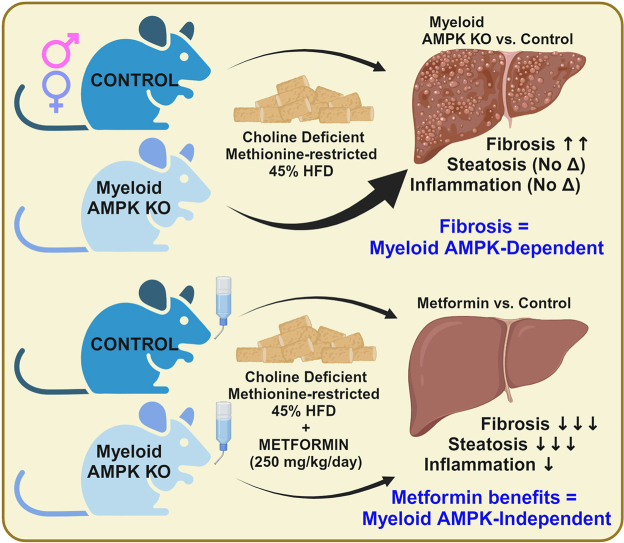

Metabolic dysfunction-associated fatty liver disease (MAFLD) captures a spectrum of diseases from simple steatosis through to non-alcoholic steatohepatitis (NASH) (1). A manifestation of metabolic syndrome, with causal ties to obesity and diabetes, the incidence of MAFLD is projected to affect over 30% of adults by 2030 (2). While simple steatosis can be considered relatively benign, lipotoxic species can accumulate in a subset of individuals, resulting in hepatocellular death and the onset of inflammation and fibrosis (i.e., NASH as well as metabolic dysfunction-associated steatohepatitis; MASH). In response to hepatic damage signals, liver-resident macrophages called Kupffer cells (KCs) secret signals that recruit monocytes as well as other immune mediators. KCs can also cause the activation of hepatic stellate cells, which produce collagen, facilitating fibrogenesis (3, 4). In recent years, the nuanced complexities of the heterogeneity of the hepatic macrophage landscape have become clearer (5). With the identification of specific macrophage subsets, many unanswered questions remain as to the plasticity of these immune populations, their activation state, and their associated metabolic programming and crosstalk.

Metabolic programming in response to nutrient and energy availability underpins the function of all cells. The AMP-activated protein kinase (AMPK) is a heterotrimeric serine/threonine kinase that signals to promote catabolic pathways and suppresses anabolic programs. In the context of metabolic dysfunction, there is extensive evidence of the protective effect of hepatocyte AMPK in both gain- and loss-of-function models (6, 7, 8). In recent years, with the use of *in vivo* models of point mutations that specifically interrogate single targets, we and others have begun to dissect the role of various AMPK signaling axes (9, 10). With an appreciation for the role of hepatocyte-specific AMPK and an understanding of the beneficial effects of activating AMPK in the liver niche, the contribution of the immune compartment in the onset and progression of NASH remains unclear.

In recent years, there have been concerted efforts to generate clinically relevant pharmacological activators of AMPK and/or to repurpose/use existing drugs to combat various chronic metabolic disorders, including MAFLD and NASH/MASH (7, 9, 11, 12, 13, 14, 15). The biguanide metformin remains one of the most widely prescribed drugs worldwide, has long been a first-line defense in the treatment of insulin resistance and type 2 diabetes, and activates AMPK signaling in the liver (16). While preclinical models have illuminated several potential mechanisms driving several of the beneficial effects of metformin (17), dose, route of administration and physiological readout are the main considerations. In the context of NASH/MASH progression, there is some clinical evidence that metformin administration is associated with improved prognosis (18, 19, 20, 21).

The liver exists as a hierarchically structured organ where spatial cellular orientation provides specialized function (22). Understanding of how metabolic programs within these cellular compartments affect and are affected by steatosis and progression to fibrosis continues to grow. Without AMPK signaling in the hematopoietic compartment during diet-induced obesity, a generalized pro-inflammatory program led to hepatic insulin resistance, in the absence of worsened steatosis (23). However, this approach failed to capture the importance of AMPK signaling in the context of liver-resident and infiltrating macrophages during disease onset and progression (5), and few studies have specifically interrogated AMPK signaling, regardless of cell-type, in the context of steatohepatitis (24, 25, 26).

Few preclinical models fully capture the translational aspects of human NASH/MASH. Rather than mirror the progression toward MASH via lengthy, nutrient-rich diets, there are approaches that aim to achieve fibrosis via non-conventional diets, which allow interrogation of pathways toward the end pathology. One such option is a methionine/choline-deficient diet, which induces steatosis and fibrosis within a short time. However, the severity of this diet coupled with confounding effects (such as weight loss) led to the development of a diet that is deficient for choline and with restricted levels of methionine in conjunction with high-fat content (CDAHFD), which has been shown to augment NASH progression without affecting weight (27). Given the role of myeloid AMPK signaling in modulating macrophage polarization in the context of adipose tissue inflammation (23), skeletal muscle regeneration (28, 29) and atherosclerosis (30, 31, 32, 33), we sought to clarify if AMPK signaling in resident and infiltrating liver macrophages was protective in a CDAHFD-induced NASH model. In male and female mice, CDAHFD-induced significant fibrosis and worsened circulating levels of liver injury markers. While indices of lipid homeostasis were unchanged, fibrosis was significantly elevated in mice lacking myeloid AMPK. Despite this baseline difference, markers of steatosis, fibrosis and inflammation were improved following metformin treatment, effects independent of myeloid AMPK signaling.

## Material and Methods

### Mice

All mice used in this study were on a C57Bl/6J background. Mice were fed a standard chow (Teklad Global 18% Protein Rodent Chow Diet T.2018.15 – Envigo/Inotivco) prior to dietary intervention and housed in a barrier facility with a 12-light/12-h dark cycle (7 pm-7 am dark). Generation of *Prkaa1*^fl/fl^ and *Prkaa2*^fl/fl^ mice have been previously described (7, 34). Double *Prkaa1/2*^fl/fl^ mice (referred to as Flox) were crossed with hemizygous Lysozyme M *Cre* (35) (LysM-*Cre*) positive mice (referred to as MacKO). Flox (*Cre* negative) and MacKO (*Cre* positive) male and female mice (11–13 weeks old) were fed a macronutrient complete low fat control diet (L-amino acid diet with 10 kcal% fat with normal methionine and choline or LFD– Research diets A06071322) or a choline-deficient, methionine-defined high fat (L-amino acid diet with 45 kcal% fat with 0.1% methionine and no added choline or CDAHFD – Research diets A06071309) for 8 weeks. Metformin (Sigma – cat. 317240) was administered in the drinking water 4 weeks into the dietary intervention at dose of 50 or 250 mg/kg/day (mpk) and replaced every 3 days (water intake was measured over 24 h and metformin stocks were diluted appropriately). Random blood glucose was measured with a glucometer prior to the tissue harvest. All animal protocols were approved by the uOttawa Animal Care Committee (BMIb-3549/BMIe-3644).

### Serum and liver lysates assays

Terminal blood was collected via cardiac puncture of the left ventricle and placed on ice. Serum was collected from clotted blood by centrifugation. Serum alanine aminotransferase (ALT), aspartate aminotransferase (AST), alkaline phosphatase (ALP), total bilirubin (TBIL), triglyceride (TG), total cholesterol as well as HDL- and LDL-cholesterol were measured using the Beckman Coulter AU480 clinical chemistry analyzer at the Pathology core at Toronto Centre for Phenogenomics. Liver was snap-frozen in liquid nitrogen and chipped on dry ice for analysis. Liver lipids were extracted using a Bligh and Dyer lipid extraction and total protein was quantified using a bicinchoninic acid assay as per the manufacturer’s instructions (Fisher – cat. PI-23225). Liver cholesterol and triglyceride were quantified from the lipid fraction with colorimetric kits (L-Type Triglyceride M – Fujifilm Wako, Infinity Cholesterol Liquid Stable Reagent – ThermoFisher) followed by normalization to total protein level in the liver (Pierce™ BCA Protein Assay Kits, ThermoFisher - 23225 which was modified with 2% SDS to reduce lipid interference) (36).

### Histology

Intact livers were perfused with PBS via the left ventricle and fixed in 30 ml of 10% neutral buffered formalin for 24 h. Following fixation, samples were transferred to 70% ethanol and processed at the University of Ottawa Louis Pelletier Histology Core Facility. Samples were paraffin-embedded, microtome sectioned and stained with H&E, Masson’s Trichrome and Picrosirius Red. Quantification of total collagen was determined using the Total Collagen Assay Kit (Abcam – ab222942), as per the manufacturer’s instructions.

### Transcript expression

PBS-perfused snap frozen livers were chipped on dry ice and homogenized in TriPure Isolation Reagent for RNA extraction (Sigma – cat. 11667165001) as per the manufacturer’s instructions. RNA was analyzed for 260/280 purity (∼2.0), equalized, and reverse transcribed (All-in-one RT Master Mix – ABM). Real-time qPCR was performed with BlasTaq™ Probe 2X qPCR Master Mix and run on Qiagen Rotor-Gene Q 2plex HRM instrument. Relative transcript expression was determined using the delta-delta Ct method, where the average Ct of the gene of interest was normalized to that of *Actb* and *Hprt* and shown relative to mice fed the LFD. Primer sequences are available in Supplemental Table S1.

### Flow cytometry

Remaining PBS-perfused livers were gently minced in 10 ml digest buffer composed of complete DMEM (Wisent – 3190005-CL with 10% FBS, 1% penicillin-streptomycin) with 0.5 mg/ml collagenase IV (Millipore Sigma – C5138) and 50 U/ml DNase I (Millipore Sigma – 1010459001). Once all tissues were collected, liver digests were allowed to warm to 37°C for 5 min, followed by shaking incubation for 20 min at 200 rpm. Following the incubation, liver digests were diluted with 25 ml of cold complete DMEM and passed through a 100 μM cell strainer. Hepatic immune cells were enriched by a series of 50 g centrifugation for depletion of hepatocytes. Finally, immune cells were pelleted at 500 g centrifugation for 5 min and collected for the following stain on ice; 30 min 1:250 Fc receptor block and 1:500 Live/dead blue, 20 min primary antibody stain (1:400 CD45.2-PerCP-Cy5.5, 1:100 F4/80-BV711, 1:600 CD11b- APC-eFluor 780, 1:400 TIM4-Alexa Fluor 647, 1:200 VSIG4-PE), and 15 min fixation with 4% paraformaldehyde. All samples were acquired on the Cytek® Aurora 5L UV-V-B-YG-R with preset optimized voltages.

### Western Blotting

PBS-perfused snap-frozen livers were chipped on dry ice and homogenized in denaturing lysis buffer (50 mM Tris-HCl pH 7.5, 150 mM NaCl, 1 mM EDTA, 0.5% Triton X-100, 0.5% NP-40, 100 μM Na_3_VO_4_, protease inhibitor cocktail). Protein concentration was determined by BCA (Pierce™ BCA Protein Assay Kits, ThermoFisher – 23225) and equalized. Following equalization, lysates were diluted in 6X SDS-PAGE loading dye and boiled at 95°C for 10 min. Protein samples were loaded twice onto 8% SDS-PAGE gels and electrophoresed at a constant 110 V for 2 h. Gels were transferred on PVDF membranes using the Trans-Blot Turbo System (Bio-Rad, 25 V, 2.5 A for 20 min) using the 5X Turbo Buffer (Bio-Rad transfer kit – 1704272). Following the semi-dry transfer, membranes were blocked with 5% BSA (in TBST) for 1 h with gentle rocking prior to overnight immunoblotting with primary antibodies made in blocking buffer. Membranes were washed with TBST for 5 min with gentle rocking, followed by immunoblotting with anti-Rb HRP-conjugated secondary antibodies, then TBST-washed again. Membranes were imaged with Clarity Western ECL substrate (Bio-Rad – 1705061) on the ImageQuant LAS4000 (GE). Antibodies are available in Supplemental Table S2.

### Statistics

GraphPad Prism software (version 9.5.1) was used for all statistical analysis. Two-way ANOVA was performed for intra-sex comparisons between LFD, CDAHFD or CDAHFD + Met, where a Tukey’s Multiple comparisons test was used to interrogate differences within treatment and genotype.

## Results

### Metformin impacts body weight and liver lipids independent of myeloid AMPK

**Fig. 1:**
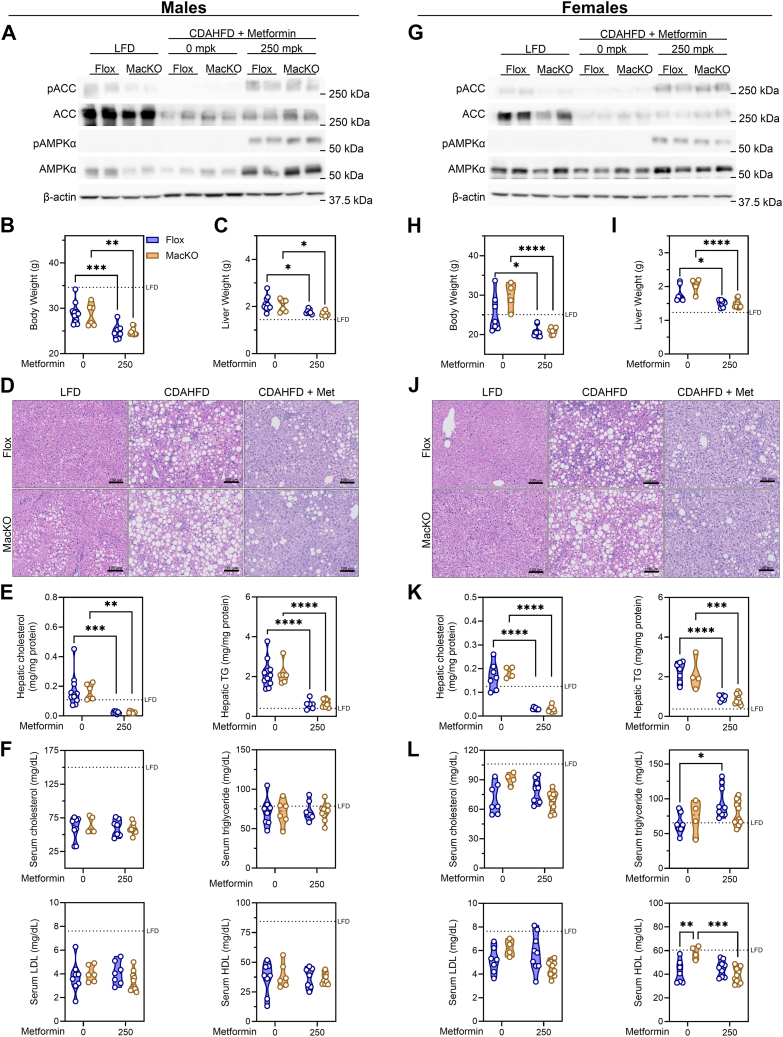
Metformin intervention improves hepatic steatosis in a myeloid AMPK-independent manner. A and G: Western blots of whole liver lysate from male and female control *Prkaa1/2*^fl/fl^ (Flox) and *Prkaa1/2*^fl/fl^/LysM-Cre^+^ (MacKO) mice fed a LFD, CDAHFD or CDAHFD + 250 mg/kg/d metformin. B and H: final body weights (g) C and I) liver weight (g) D and J) representative H&E-stained liver sections (20x magnification, 100 μm scale bar). E and K: hepatic cholesterol and triglyceride normalized to total protein. F and L: serum total cholesterol, total triglycerides, LDL-cholesterol and HDL-cholesterol. Data were analyzed by Two-way ANOVA with Tukey’s test for multiple comparisons where ∗, ∗∗, ∗∗∗ and ∗∗∗∗ represent *P* < 0.05, *P* < 0.01, *P* < 0.001, and *P* < 0.0001, respectively. The geometric mean of LFD Flox controls is represented by the hashed lines.

The protective effect of hepatocyte AMPK against MAFLD progression has been clearly demonstrated in genetic and pharmacological studies. To investigate the significance of AMPK in all bone marrow-derived and tissue-resident myeloid cells, we used male and female Flox and MacKO mice, and induced NASH over the course of 8 weeks using a CDAHFD, which is known to produce hepatic steatosis, inflammation, and F2 fibrosis (37). When fed an LFD, there were no differences between genotypes in either sex (Supplemental Table S3). As expected, compared to LFD-fed mice, CDAHFD-fed males maintained their body weight, whereas females gained similar weight as LFD controls (Supplemental Fig. S1, A, B). Numerous models of MAFLD/NASH, including a version of CDAHFD, have observed reduced AMPK activity in the total liver homogenate (7, 24). Therefore, to address this general suppression, we reactivated AMPK by introducing metformin at a dose of 50 or 250 mpk administered in the drinking water in the latter 4-weeks of the study. We sought to investigate whether clinically relevant doses of oral metformin had effects on NASH progression and whether myeloid-specific AMPK signaling was involved. Although mice treated with metformin at 50 mpk showed no sign of AMPK signaling to its downstream target acetyl-CoA carboxylase (ACC) at the study endpoint (Supplemental Fig. S1C), 250 mpk metformin treatment resulted in elevated phosphorylation of both ACC and AMPK (Fig. 1A, G). Moreover, reactivation of AMPK signaling by higher dose metformin was observed despite clear downregulation of ACC protein levels. Given that lower dosing of metformin (50 mpk) failed to activate AMPK signaling, we focused our characterization of NASH measures on the group receiving the higher, yet still physiological (250 mpk) dose of metformin (38, 39).

There were no genotype differences in body weight after 8 weeks of CDAHFD feeding and intervention with 250 mpk metformin lowered body weight regardless of genotype and sex (Fig. 1B, H). Although the CDAHFD model does not induce obesity or hyperglycemia, liver weight was increased in both males and females independent of myeloid AMPK, an effect normalized by 250 mpk metformin treatment (Fig. 1C, I). Given the effects on total body and liver weight, liver weight as a percent of the total weight revealed no genotype or metformin-associated differences (Supplemental Fig. S2). Despite this normalization, hepatic macro- and micro-vesicular steatosis observed in both male and female mice (Fig. 1D, E, J, K), corroborated biochemical assessment of total liver cholesterol and triglyceride (TG), which were significantly elevated by the CDAHFD, and dramatically reduced in response to metformin treatment independent of genotype and sex. One of the mechanisms by which CDAHFD expedites NASH progression is the disruption of lipoprotein production and secretion as choline is a critical for this pathway (27). As expected, compared to control-fed mice, CDAHFD-fed mice had lower circulating total and LDL-cholesterol (Fig. 1F, L), without changes due to genotype or metformin treatment. However, while there were no differences in circulating levels of triglycerides in any of the male groups, metformin significantly increased serum triglyceride levels in female mice in a myeloid AMPK-dependent manner (Fig. 1L). Moreover, HDL-cholesterol levels were significantly elevated upon myeloid AMPK deletion in female mice, although this was reduced by metformin independent of myeloid AMPK signaling (Fig. 1L). Overall, deletion of myeloid AMPK had minimal influence on circulating or hepatic markers of steatosis; however, intervention with metformin significantly improved these measures in all groups.

### Hepatic fibrosis is elevated by myeloid AMPK disruption and ameliorated by metformin treatment

**Fig. 2:**
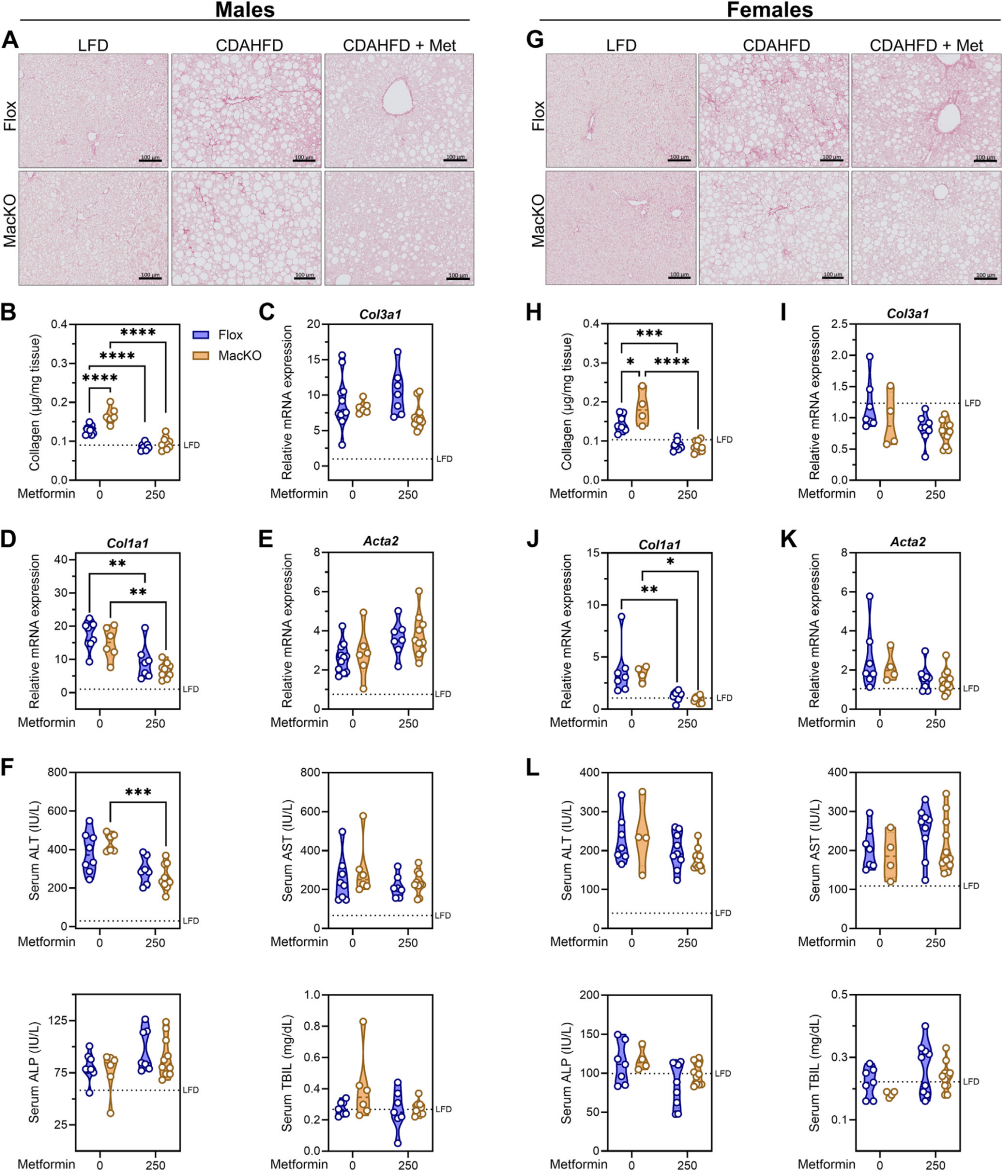
Myeloid AMPK restricts fibrosis but is not critical for metformin-associated improvements. A and G: representative Picrosirius Red-stained liver sections (20x magnification, 100 μm scale bar) of male and female control *Prkaa1/2*^fl/fl^ (Flox) and *Prkaa1/2*^fl/fl^/LysM-Cre^+^ (MacKO) mice fed a CDAHFD or CDAHFD + 250 mg/kg/d metformin. B and H: total collagen quantification. C–E and I–K: hepatic mRNA transcript levels of markers of extracellular matrix-regulating transcripts (*Col3a1*, *Col1a1*, *Acta2*). F and L: serum levels of liver injury markers (alanine aminotransferase (ALT), aspartate aminotransferase (AST), alkaline phosphatase (ALP), and total bilirubin (TBIL)). Transcript expression was normalized to the average expression of *Actb* and *Hprt* and expressed relative to LFD-fed Flox mice. Data were analyzed by Two-way ANOVA with Tukey’s test for multiple comparisons where ∗, ∗∗, ∗∗∗, and ∗∗∗∗ represent *P* < 0.05, *P* < 0.01, *P* < 0.001, and *P* < 0.0001, respectively. The geometric mean of LFD Flox controls is represented by a hashed line.

As expected, feeding mice a CDAHFD for 8 weeks increased hepatic fibrosis as shown by Picrosirius red and biochemical quantification of total collagen in comparison to LFD control (Fig. 2A, B, G, H). Interestingly, disruption of myeloid AMPK activity significantly augmented the amount of hepatic fibrosis in both male or female mice (Fig. 2B, H). However, despite this genotype effect, metformin intervention completely normalized collagen levels in the liver of both genotypes, independent of sex (Fig. 2B, H). At the transcriptional level, there were no measurable differences in the expression of collagen peptide transcripts (*Col3a1, Col1a1*) to explain genotype differences in males or females; however, the metformin treatment was associated with significantly lower levels of *Col1a1*, but not *Col3a1*, with no changes in the expression of *Acta2* as a marker of hepatic stellate cell activation (Fig. 2C–E, I–K). Finally, circulating levels of liver injury markers indicated a clear effect of CDAHFD-induced changes, there were no appreciable changes attributable to myeloid AMPK or metformin treatment (Fig. 2F, L).

### Metformin treatment, but not deletion of myeloid AMPK, affects markers of hepatic inflammation

**Fig. 3:**
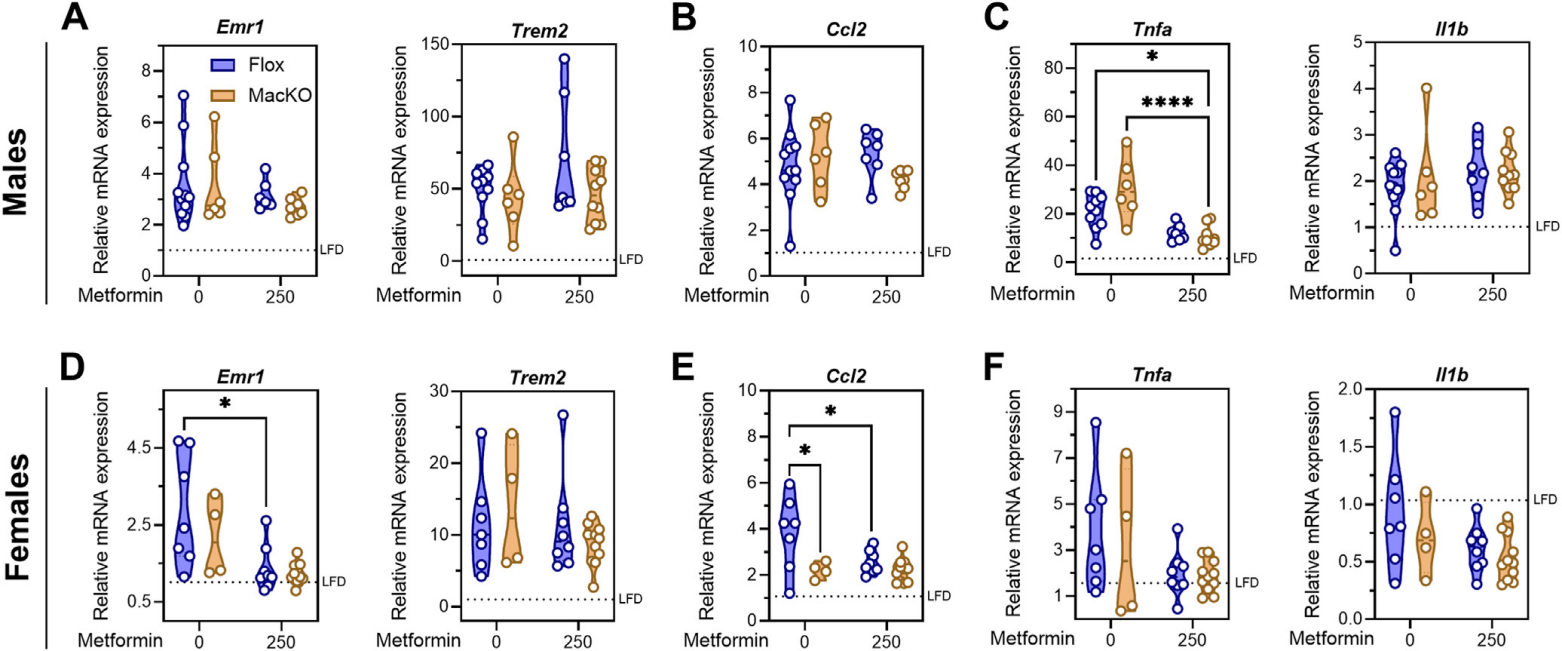
Metformin intervention reduces some markers of hepatic inflammation in a myeloid AMPK-independent manner. A and D: Hepatic mRNA transcript levels of macrophages markers (*Emr1*, *Trem2*) from male and female control *Prkaa1/2*^fl/fl^ (Flox) and *Prkaa1/2*^fl/fl^/LysM-Cre^+^ (MacKO) mice fed a CDAHFD or CDAHFD + 250 mg/kg/d metformin. B and E: the marker of monocyte recruitment (*Ccl2*) and (C and F) inflammatory cytokines (*Tnf*, *Il1b*). Transcript expression was normalized to the average expression of *Actb* and *Hprt* and shown relative to LFD-fed Flox mice. Data were analyzed by Two-way ANOVA with Tukey’s test for multiple comparisons where ∗ and ∗∗∗∗ represent *P* < 0.05 and *P* < 0.0001, respectively. The geometric mean of LFD Flox controls is represented by a hashed line.

Myeloid AMPK signaling restricted fibrosis development in the CDAHFD model; however, systemic metformin treatment ameliorated all diet-associated effects, independent of genotype. We were next interested in potential differences in markers of macrophage infiltration and inflammatory signaling. In the setting of NASH, bone marrow-derived monocytes differentiate into different macrophage subsets, including Trem2^+^ lipid-associated macrophages (LAMs) that can form hepatic crown-like structures (40, 41, 42, 43). In agreement with this, we observed increased transcript expression of *Emr1* and *Trem2* in CDAHFD males (Fig. 3A), while only *Trem2* expression was increased in female mice (Fig. 3D). This is suggestive of high macrophage content in the liver derived from infiltrating monocytes, which was unaffected by the presence of myeloid AMPK but tended to be lower in females treated with systemic metformin. Moreover, there was augmented expression of monocyte chemokine *Ccl2*, which may signify higher monocyte infiltration in CDAHFD-fed males (Fig. 3B). The transcript expression of *Ccl2* was significantly lower in female MacKO mice, and metformin was subsequently able to lower levels in control mice (Fig. 3E). Finally, we observed a robust increase the expression of *Tnfa* (though not *Il1b*) in CDAHFD-fed males, which was significantly lower in metformin-treated mice, independent of genotype (Fig. 3C). The transcriptional response of *Tnfa* and *Il1b* in CDAHFD-fed females was less drastic compared to males and showed no dependence on myeloid AMPK signaling and only trends towards decreased levels in response to metformin (Fig. 3F).

## Discussion

We provide evidence that endogenous myeloid AMPK signaling restricts the development of fibrosis during CDAHFD-induced NASH progression but that AMPK signaling in these cells is dispensable for metformin-associated improvements in markers of NASH. There is extensive evidence of the protective effects of activating hepatocyte-specific or hepatocellular AMPK activity in various dietary models of fatty liver disease. Recently, our understanding of this role was extended to include limiting cell death through the inhibition of caspase-6 (24). While many studies focused on the role of AMPK in hepatocytes specifically or in the overall liver environment, few have assessed the importance of AMPK signaling within the hepatic immune compartment. Deletion of the AMPK β1 subunit from the hematopoietic compartment worsens liver inflammation and increases hepatic immune infiltration in male mice fed an HFD (23). In contrast, LysM-mediated deletion of the AMPK α1 subunit was shown to diminish monocyte recruitment and macrophage infiltration under high-fat feeding conditions, which was accompanied by worsened steatosis and insulin resistance in a mix of male and female mice (33). Our data suggest that removing AMPK signaling in myeloid cells has little consequence on the degree of steatosis or inflammation, but significantly impacted the level of fibrosis in male and female mice. Previous work showed that whole-body deletion of AMPK α1 in female mice failed to affect CCl_4_-induced hepatic fibrosis (44). However, it is important to consider that our observations may be specific to the strain of mice (C57Bl/6J), dietary intervention (CDAHFD) and duration of the study (8 weeks).

KCs are key players in the initiation of inflammation, immune infiltration as well as communication with damaged hepatocytes, and activation of hepatic stellate cells (5). However, NASH macrophage populations are more heterogeneous than the simple distinction made between tissue-resident and infiltrating macrophages. KCs can be segmented by their expression of endothelial cell-specific adhesion molecules and NASH-induced expression of CD36, which plays an important role in liver oxidative stress (45). Infiltrating macrophages that arise during NASH can also be subdivided into LAMs and macrophages that repopulate the KC niche (MoKCs) (41, 42, 43). Given the complex heterogeneity of hepatic macrophages during NASH, our goal was to assess the importance of AMPK in all macrophages in the hepatic microenvironment during NASH progression.

To target AMPK signaling specifically, we chose to use the *LysM* promoter for the expression of *Cre*. Our model makes use of a double deletion of both catalytic subunits of AMPK (*Prkaa1* and *Prkaa2*). While the α1 subunit is predominantly expressed in immune subsets (46), we reasoned that dual deletion would ensure complete disruption of AMPK signaling in LysM-expressing cells. To this end, monocytes and differentiated macrophages represent the majority of cells that undergo LysM-mediated recombination and AMPKα1/α2 deletion; however, we cannot rule out the potential targeting of granulocytes such as neutrophils (47). Neutrophils not only contribute to fibrogenesis indirectly, but infiltration of this population also significantly contributes to the production of inflammatory cytokines (48). Despite this, LysM-mediated recombination has been shown to be high for both KCs and MoKCs (49). However, *Cre* recombination efficiency may not be equal amongst different macrophage subsets within the hepatic environment, both under normal conditions and NASH (49). We have previously validated the deletion of AMPK signaling in thioglycolate-induced peritoneal macrophages, which represent recently differentiated monocytes and residential peritoneal macrophages (29, 30). However, there may be residual AMPK expression in infiltrating macrophage subsets such as LAMs, a macrophage population that forms hepatic crown-like structures, a key histological feature of NASH (41, 42, 43). Importantly, our study design did not integrate control mice that express only *Cre* recombinase. Therefore, we cannot rule out the possibility that fibrosis observed in LysM-*Cre* positive mice is not due to *Cre*-mediated effects.

To our knowledge, this is the first investigation into the role of myeloid-specific AMPK signaling during the onset and progression of NASH and chronic low-grade inflammation in the liver. Without changes in hepatic steatosis, deletion of AMPK in myeloid cells significantly increased collagen deposition in both male and female mice. Interestingly, there were no changes in collagen transcripts at the end of the study, suggesting there could be discordance between transcript and collagen peptide formation or potential differences in collagen breakdown (ie, an inhibition of breakdown) in MacKO livers. We recognize that characterization of the hepatic macrophage populations between LFD and CDAHFD, as well as with and without AMPK signaling would have added to our overall understanding as we cannot rule out the potential that frequencies of resident KCs and infiltrating MoKCs macrophages may be different between genotypes. We confirmed that 8 weeks of CDAHFD feeding resulted in KC depletion and replacement by MoKCs (Supplemental Fig. S3); therefore, we cannot rule out the potential importance of AMPK signaling in KCs at earlier stages of this NASH model (50). Therefore, use of a KC-specific Cre system (*Clec4f*) would restrict deletion of AMPK to resident KCs and MoKCs that experience an induction of *Clec4f* expression (51). However, future studies warrant longitudinal analysis of KC metabolism and activation under a NASH-induced diet as there may be key differences during the onset and progression with this model.

There are continuing efforts to develop a translationally relevant mouse model to mirror human NASH. We and others have characterized the effect of various dietary interventions coupled with thermoneutral housing (∼29°C) to promote the progression toward hepatic fibrosis (52, 53, 54, 55, 56), which typically ranges from 16 to 24 weeks. To accelerate fibrogenesis, we chose to introduce a CDAHFD, which produces F2 fibrosis after 8 weeks. As expected, we observed decreased lipoprotein secretion when compared to LFD-fed mice. This effect is mainly mediated by choline deficiency, which results in a high degree of steatosis in combination with a high fat content (37). Moreover, in contrast to a methionine/choline-deficient diet, the mice do not experience weight loss or a change in blood glucose. Importantly, our results confirm that female C57Bl/6J mice remain sensitive to a degree of NASH induction.

Numerous studies have reported reduced liver AMPK activity (representing the liver environment, not just hepatocytes) in various dietary models of MAFLD and NASH (7, 24). We chose to introduce the systemic delivery of metformin for the reactivation of AMPK. Metformin represents the historical first-line defense in the treatment of type 2 diabetes (among other chronic conditions) and has a storied history given the initial observations that it activates AMPK (16, 17). While the AMPK-activating and therapeutic mechanisms of AMPK remain to be fully understood (or demonstrated clinically in certain contexts), preclinical studies have shown consistent reductions in hepatic steatosis in HFD-fed mice through AMPK-dependent and independent mechanisms (17). However, its efficacy in preventing/ameliorating the progression of NASH is less clear. Given the importance of appropriate dose and delivery of metformin in preclinical studies, and since renal expression of organic cation transporters is known to be down-regulated by choline deficiency (potentially increasing local and circulating levels of metformin), we initially chose continuous oral delivery of a low dose (50 mpk) of metformin in the drinking water. While it was recently reported that low-dose metformin activates lysosomal AMPK via the PEN2-ATP6AP1 axis, thereby reducing hepatic steatosis in an HFD model (57), we did not observe indicators that AMPK was activated at the study endpoint (Supplemental Fig. S1C). While we may have failed to accurately capture AMPK activation, importantly, there were no observable changes to steatosis, fibrosis or inflammation at this dose (data not shown), indicating that low (50 mpk in the water) dose metformin dose did not affect indicators of NASH progression in the CDAHFD model. Therefore, we opted to intervene with a higher dose of metformin that was still within a physiological range (38, 39). It was clear that all metformin-associated changes were independent of myeloid AMPK, which strongly suggests that AMPK signaling in hepatocytes or other cell types, or potentially AMPK-independent mechanisms were responsible for the overall improvement. An important confounding factor that may frame our observations is that the dose of metformin resulted in a significantly lower final body weight, which may have contributed to the anti-steatotic and anti-fibrotic effects. In the future, it will be interesting to test metformin against preventative and intervention dosing of a specific and direct AMPK activator like PXL770 (25) in cell-specific AMPK null models to fully dissect the cell type and signaling that are important during the onset and progression of NASH.

AMPK signaling governs several metabolic pathways, across cell type and circumstance. Macrophage AMPK maintains physiological homeostasis by regulating lipid metabolism and inflammatory signaling. When fed a CDAHFD, myeloid AMPK limits fibrosis during NASH progression in male and female mice. However, while metformin treatment dramatically improved markers of hepatic steatosis, fibrosis, and, to a lesser extent, inflammation, these effects were independent of myeloid AMPK. The extent to which AMPK signaling can be leveraged to reprogram resident and infiltrating macrophages to improve NASH prognosis remains to be tested and may represent an additive therapeutic benefit.

## Data Availability

Data used in this study are available in the article and the supplement, or from the corresponding author upon request.

## Supplemental data

This article contains supplemental data.

## Conflict of interest

The authors declare that they have no known competing financial interests or personal relationships that could have appeared to influence the work reported in this paper.
