# Supplementary material for: Myeloid AMPK signaling restricts fibrosis but is not required for metformin improvements during CDAHFD-induced NASH in mice
Source: J Lipid Res. 2024 May 17;65(6):100564. doi: 10.1016/j.jlr.2024.100564 (PMC11222943; doi:10.1016/j.jlr.2024.100564)
Supplement: Supplemental Figure [file mmc1.docx]

**Supplemental Figures**

**
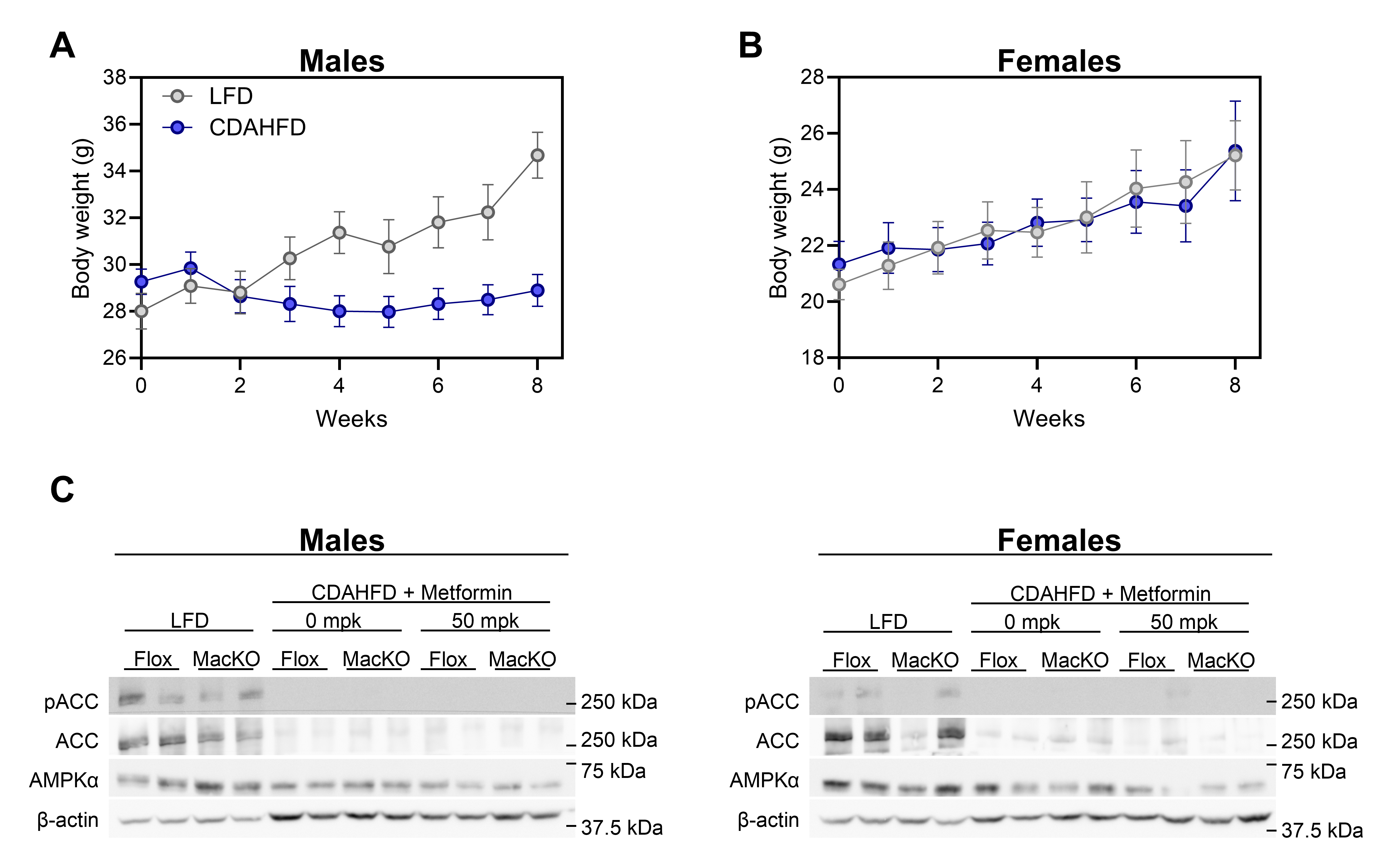
**

**Figure S1. CDAHFD does not induce weight lose in male or female mice and low-dose metformin does not activate liver AMPK. A/B)**Weekly body weights of Flox and MacKO (combined) male and female mice fed a LFD or CDAHFD. **C**) Western blots of whole liver lysate from male and female control *Prkaa1/2*^fl/fl^ (Flox) and *Prkaa1/2*^fl/fl^/LysM-Cre^+^ (MacKO) mice fed an LFD, CDAHFD or CDAHFD + 50 mg/kg/d metformin.


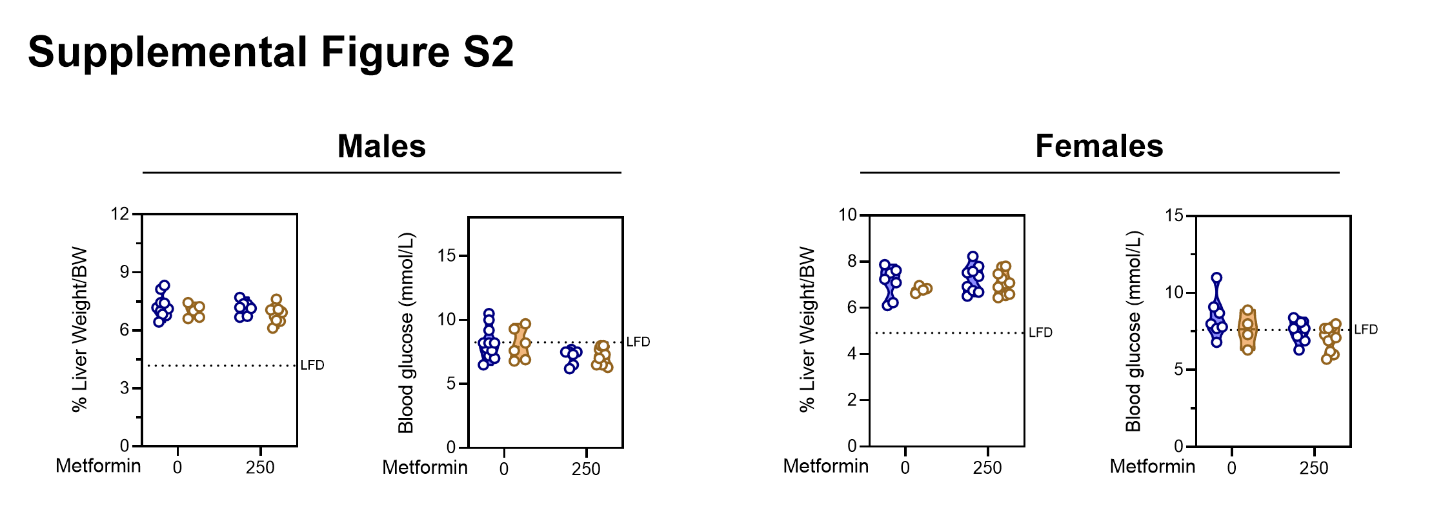


**Figure S2. Metformin does not affect normalized liver weight or blood glucose.** Normalized liver weights and random blood glucose from male and female control *Prkaa1/2*^fl/fl^ (Flox) and *Prkaa1/2*^fl/fl^/LysM-Cre^+^ (MacKO) mice fed a LFD, CDAHFD or CDAHFD + 250 mg/kg/d metformin,

**
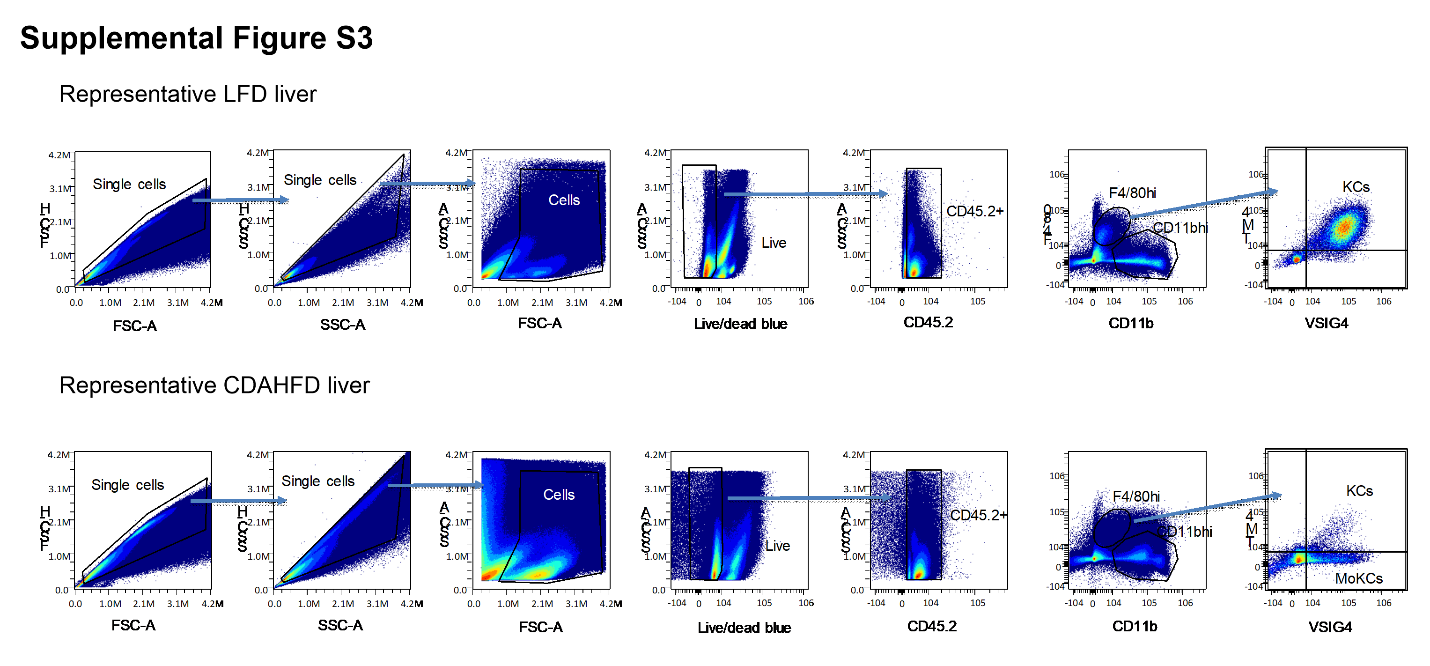
**

**Figure S3. Resident macrophage populations disappear after a CDAHFD.** Representative hepatic immune flow plots of male mice fed an LFD or CDAHFD. Gating strategy showing KCs (TIM4^+^ VSIG4^+^) and MoKCs (TIM4^-^ VSIG4^+^) from F4/80^hi^ CD11b^lo^ CD45^+^ live single cells.
