# Supplementary material for: Myeloid AMPK signaling restricts fibrosis but is not required for metformin improvements during CDAHFD-induced NASH in mice
Source: J Lipid Res. 2024 May 17;65(6):100564. doi: 10.1016/j.jlr.2024.100564 (PMC11222943; doi:10.1016/j.jlr.2024.100564)
Supplement: Supplemental Tables [file mmc2.docx]

**Table S1 – Primer sequences**

| **Gene** | **Forward primer** | **Reverse primer** |
| --- | --- | --- |
| *Col3a1* | TCTGGAAGCCAGAACCATGT | AATGGGATCTCTGGGTTGGG |
| *Col1a1* | GCTCCTCTTAGGGGCCACT | CACGTCTCACCATTGGGG |
| *Acta2* | GTCCCAGACATCAGGGAGTAA | TCGGATACTTCAGCGTCAGGA |
| *Emr1* | GCATCATGGCATACCTGTTC | GAGCTAAGGTCAGTCTTCCT |
| *Trem2* | CTGGAACCGTCACCATCACTC | CGAAACTCGATGACTCCTCGG |
| *Ccl2* | TTAAAAACCTGGATCGGAACCAA | GCATTAGCTTCAGATTTACGGGT |
| *Tnfa* | CCCTCACACTCAGATCATCTTCT | GCTACGACGTGGGCTACAG |
| *Il1b* | GCAACTGTTCCTGAACTCAACT | ATCTTTTGGGGTCCGTCAACT |
| *Actb* | GGCTGTATTCCCCTCCATGG | CCAGTTGGTAACAATGCCATGT |
| *Hprt* | TCAGTCAACGGGGGACATAAA | GGGGCTGTACTGCTTAACCAG |

**Table S2 – Western blot antibodies**

| **Target protein** | **Antibodies** | **Supplier and Catalog No.** |
| --- | --- | --- |
| AMPKα | AMPKα (D63G4) Rabbit mAb | Cell Signaling  #5832 |
| pAMPK T172 | Phospho-AMPKα (Thr172) (40H9) Rabbit mAb | Cell Signaling #2535 |
| ACC | Acetyl-CoA Carboxylase (C83B10) Rabbit mAb | Cell Signaling  #3676 |
| pACC S79 | Phospho-Acetyl-CoA Carboxylase (Ser79) (D7D11) Rabbit mAb | Cell Signaling #11818 |
| β-actin | β-Actin (13E5) Rabbit mAb (HRP Conjugate) | Cell Signaling #5125 |
| Rabbit IgG | Anti-rabbit IgG, HRP-linked | Cell Signaling # 7074 |

**Table S3 – LFD genotype comparisons**

| **Readout** | **Females Flox (mean ± SD)** | **Females MacKO (mean ± SD)** | **Males Flox (mean ± SD)** | **Males MacKO (mean ± SD)** |
| --- | --- | --- | --- | --- |
| Body Weight (g) | 25.21± 3.03 | 23.03± 0.91 | 34.68± 2.20 | 34.82± 2.15 |
| Liver Weight (g) | 1.25±0.26 | 1.44±0.57 | 1.46±0.26 | 1.56±0.06 |
| Hepatic cholesterol (mg/mg protein) | 0.15±0.09 | 0.18±0.03 | 0.12±0.05 | 0.11±0.04 |
| Hepatic triglyceride (mg/mg protein) | 0. 44±0.29 | 0.51±0.41 | 0.41±0.12 | 0.55±0.25 |
| Serum cholesterol (mg/dL) | 106.65±12.93 | 72.57±46.42 | 150.23±9.79 | 136.33±52.37 |
| Serum TG (mg/dL) | 68.43±21.59 | 51.07±17.12 | 78.88±7.99 | 77.77±14.81 |
| Serum HDL (mg/dL) | 60.75±7.54 | 36.50±17.76 | 84.45±1.78 | 79.40±23,00 |
| Serum LDL (mg/dL) | 7.67±0.82 | 5.56±2.92 | 7.65±0.79 | 7.87±4.00 |
| Serum ALT (IU/dL) | 40.63±10.67 | 151.13±155.22 | 29.95±4.31 | 47.10±11.13 |
| Serum AST (IU/dL) | 129.05±95.10 | 284.03±290.72 | 67.23±11.47 | 78.47±6.52 |
| Serum ALP (IU/dL) | 100.20±13.21 | 100.2±53.04 | 58.75±7.85 | 56.50±5.57 |
| Serum TBIL (IU/dL) | 0.23±0.07 | 0.17±0.06 | 0.33±0.26 | 0.48±0.52 |
